# Pregnancy-Associated Breast Cancer: What Radiologists Must Know

**DOI:** 10.7759/cureus.10343

**Published:** 2020-09-09

**Authors:** Dafne Soto-Trujillo, Lourdes N Santos Aragón, Yukiyoshi Kimura

**Affiliations:** 1 Radiology, Centro Medico ABC, Mexico City, MEX; 2 Breast Imaging, Centro Medico ABC, Mexico City, MEX; 3 Radiology, Instituto Nacional de Ciencias Medicas y Nutricion Salvador Zubiran, Mexico City, MEX

**Keywords:** breast cancer, pregnancy

## Abstract

Pregnancy-associated breast cancer (PABC) is defined as breast cancer diagnosed during pregnancy, in the first year postpartum, or during breastfeeding. Imaging techniques play a significant role in the diagnosis of PABC. It is a challenging diagnosis since physiological changes seen in pregnancy and breastfeeding may limit clinical suspicion and imaging utility. The patient's well-being and the fetus must be taken into consideration for diagnosis and treatment.

## Introduction and background

Epidemiology

Pregnancy-associated breast cancer (PABC) is defined as breast cancer diagnosed during pregnancy, in the first year postpartum, or during breastfeeding [1]. PABC corresponds to less than 3%-5% of all cases. However, it represents up to 20% of breast cancers in women up to 30 years [2]. The reported incidence of PABC is 15-35 per 100,000 deliveries [3]. Incidence is lower during pregnancy, with 2/3 of all cases are detected in the first six months postpartum. The patients' average age is 32-34 years, which is lower than the overall incidence of breast cancer. There is a high risk associated in patients with family history and BRCA mutations. Most patients do not have a family history of breast cancer [4]. 

Pathologic features

As in non-pregnant women, ductal infiltrating adenocarcinoma is the most common histological type, with a lower expression of hormone receptor (estrogen and progesterone) [5], HER‐2/neu expression is unclear since studies have found negative expression during pregnancy and lactation and positive expression after delivery or cessation of lactation. It is believed to be secondary to high circulating estrogen levels during pregnancy that downregulate estrogen receptors' expression in some cell lines [6]. There is a higher incidence of poorly differentiated and inflammatory breast cancer in PABC compared to non-pregnant women [7].

Clinical manifestations

Patients present with a painless palpable mass (90%) or thickening of the skin. Lymph node involvement and inflammatory changes are expected at diagnosis. Diagnosis is usually delayed due to physiological changes related to pregnancy that overlook breast masses [4]. The average tumor size at diagnosis is 3.5 cm, and lymph node metastasis is frequent. Delay in diagnosis is due to normal breast hyperplasia in pregnancy that may mask palpable masses [7]. A delay in imaging studies and invasive procedures for diagnosis based on fear of the fetus's welfare may delay management. 

## Review

Imaging in pregnant women 

Ultrasound

Ultrasound is the initial image modality with a reported sensitivity of up to 100% and a negative predictive value of up to 100% [8]. Since there is an increase in the gland's nodularity associated with pregnancy, any mass that persists for more than two to four weeks should raise suspicion for malignancy and must be assessed initially by this imaging method. 

**Figure 1:**
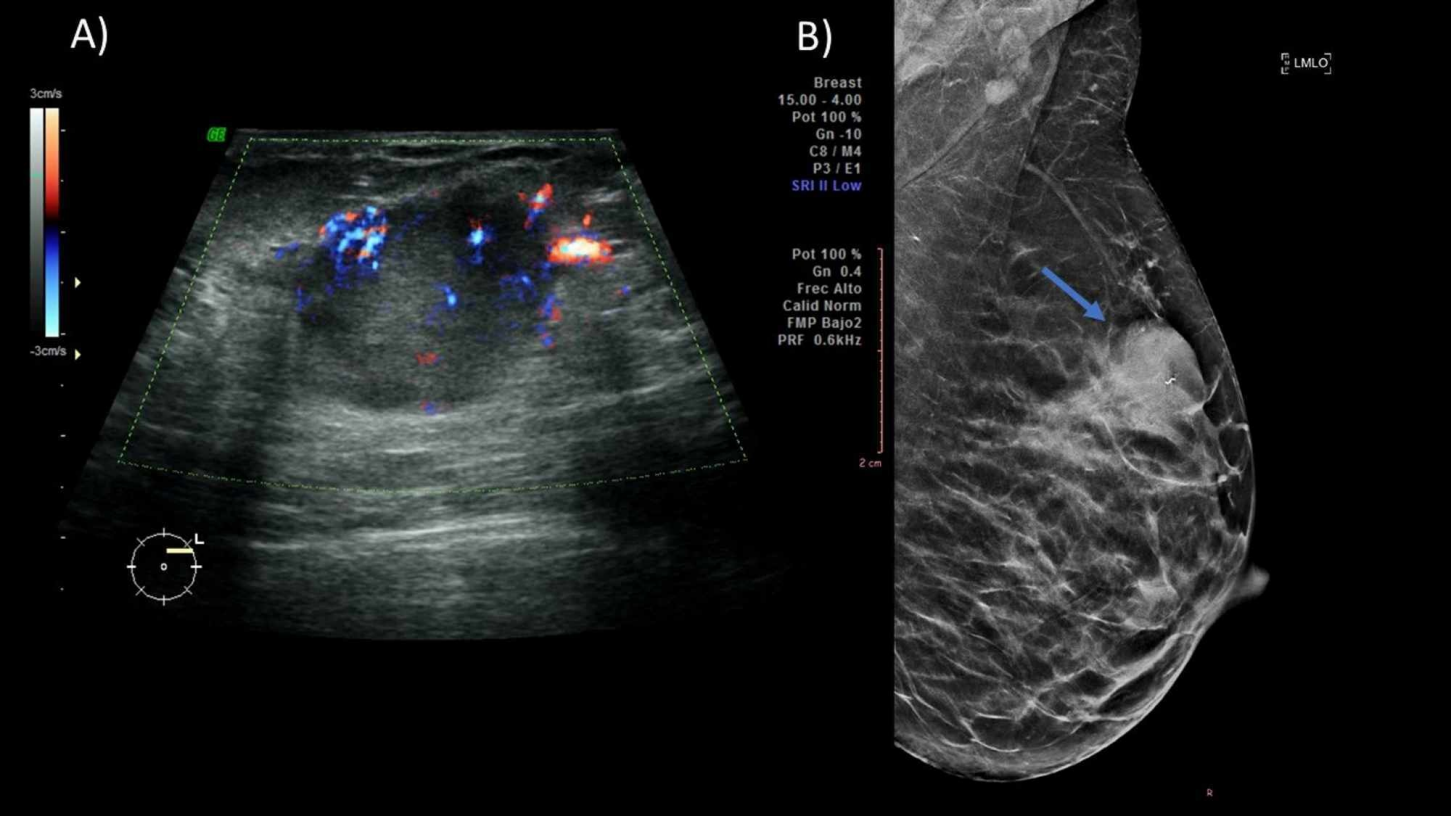
A 36-year-old female patient in her second trimester with a palpable mass in the left breast that presents growth in the last three months. (A) Ultrasound showing at 2:00 a hypoechoic nodule with micronodulated margins of 2.8 cm, and Doppler showing peripheral vascularity. (B) MLO projection of the left breast showing a hyperdense nodule of irregular margins (blue arrow) that causes skin retraction. A percutaneous biopsy was performed, with a triple-negative high-grade infiltrating ductal carcinoma result. MLO, mediolateral oblique

PABC is observed as hypoechoic masses with cystic components due to associated central necrosis. The borders are irregular and may have a posterior acoustic shadow or echogenic halos [9] (Figure 1). When circumscribed margins, oval shape, and parallel orientation are seen in a suspicious lesion, benign pathology may be suggested, and no further evaluation is necessary [10]. ​​​

Mammography

**Figure 2:**
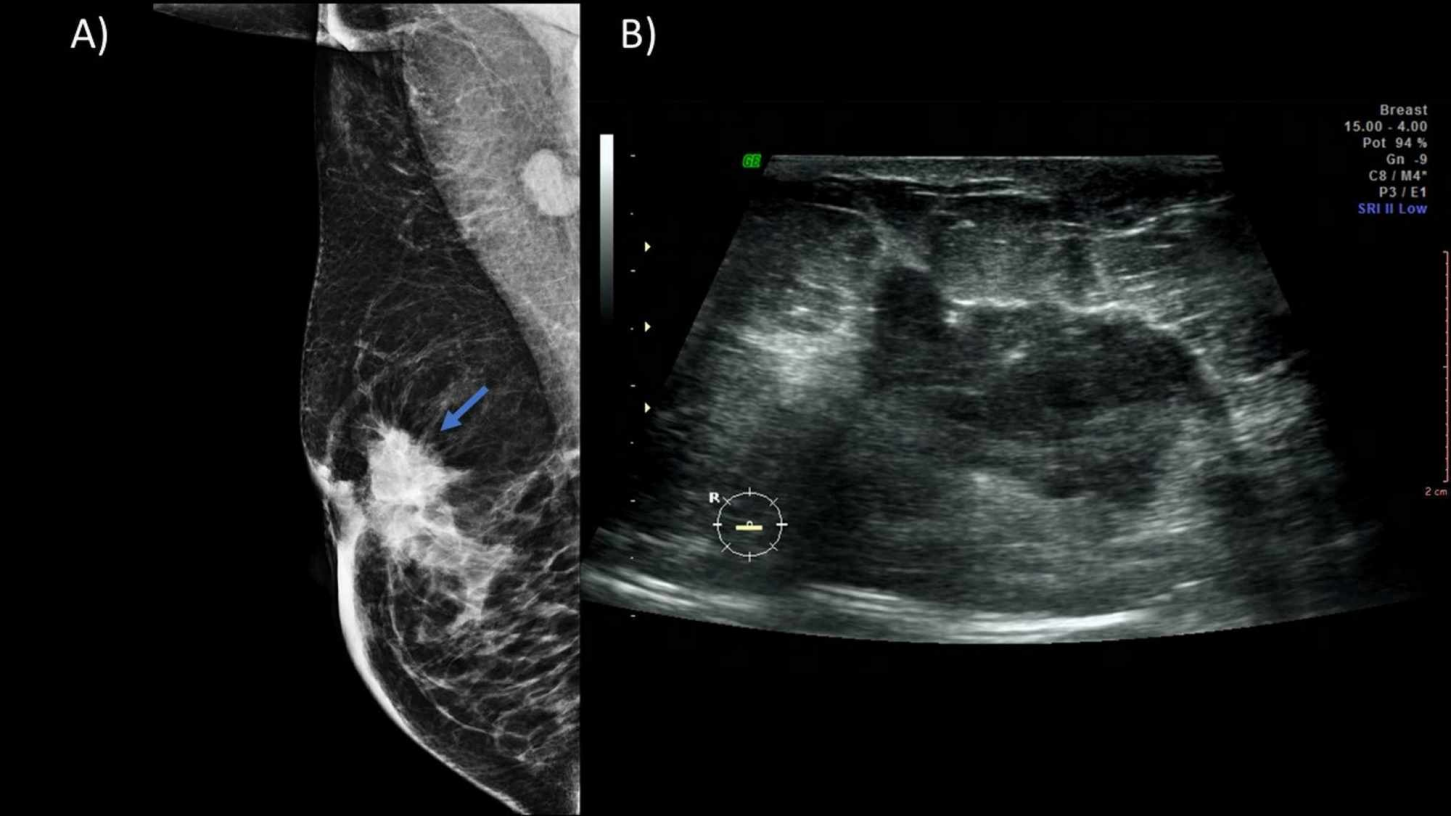
A 35-year-old patient in the immediate puerperium with a palpable nodule in the right breast. (A) MLO projection of the right breast showing a hyperdense nodule (blue arrow) in the retroareolar region of spiculated margins that conditions skin thickening and retraction. (B) Ultrasound showing a hypoechoic nodule of 5 cm, spiculated borders in the retroareolar region. A percutaneous biopsy was performed with a moderately differentiated infiltrating ductal carcinoma result. MLO, mediolateral oblique

Bilateral mammography is recommended in patients with clinical suspicion. The sensitivity of mammography in PABC is 78%-90% (even in dense breasts). Radiation to the fetus is minimal, with an estimated dose of less than 0.03 uGy; therefore, two basic projections are encouraged [10]. Suspicion findings include nodules, masses, microcalcifications, asymmetries, distortions in the architecture, or thickening of the skin (Figure 2).

Mammography is beneficial in evaluating microcalcifications, which are not seen in ultrasound evaluation and are determinant to evaluate the disease extent (multifocal multicentric and contralateral disease) [9]. Routine screening mammography must be continued in patients over 40 years three months after lactation cessation.

Magnetic Resonance

The use of MRI in patients with PABC is not used routinely recommended. It is reserved for patients in whom the cost-benefit is evident [10]. The contrasted agents are classified as type C drugs, according to the FDA. Gadolinium crosses the fetus-placental barrier and can be eliminated by the fetal kidneys. MRI is safe in breastfeeding patients since gadolinium has a minimal excretion through milk (rate of 0.0004%) [11]. According to the American College of Radiology Guidelines, breastfeeding suspension is not required [12].

**Figure 3:**
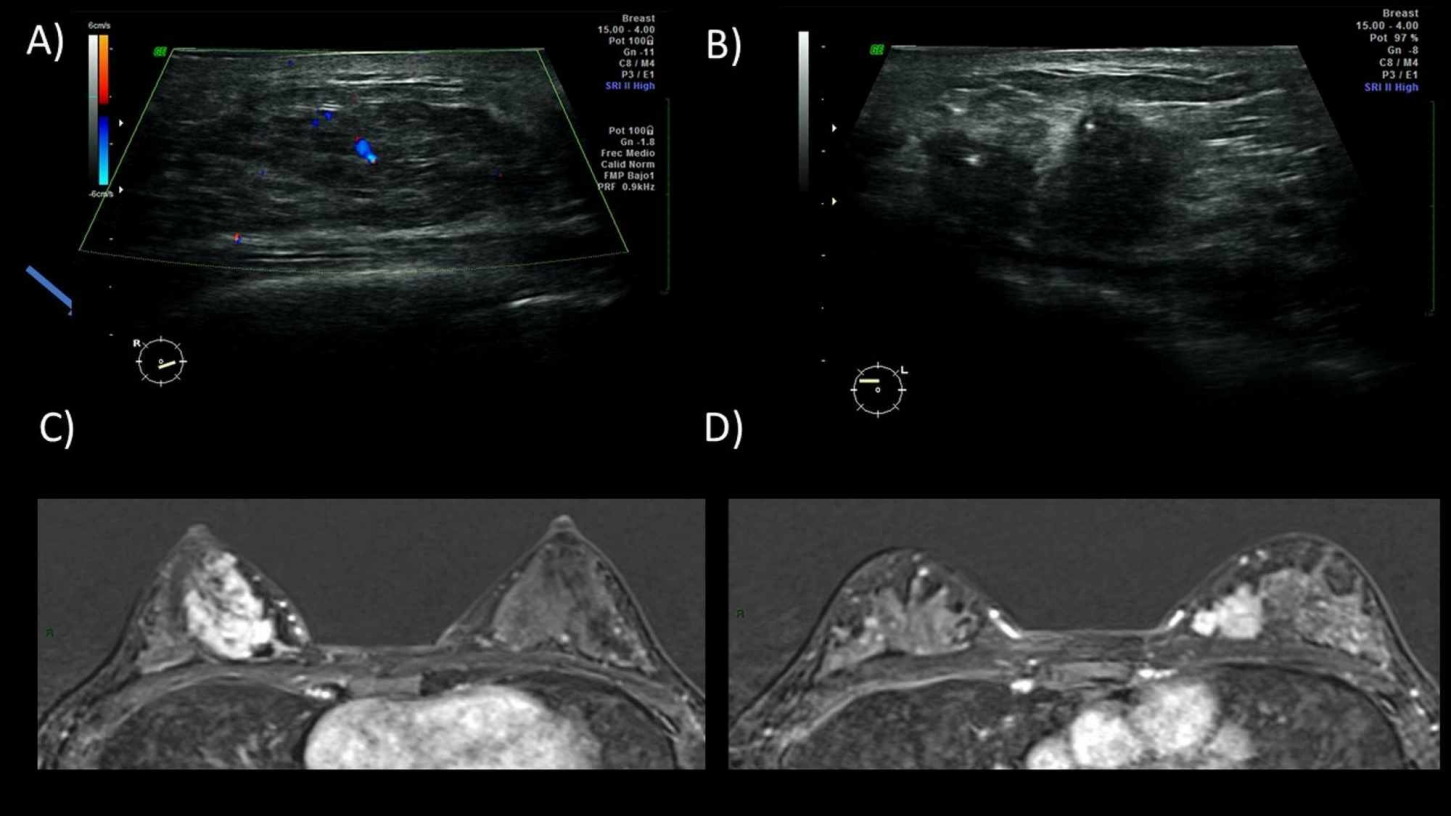
A 38-year-old female patient in puerperium and lactation felt nodules on both breasts since her first trimester. Ultrasound was performed. (A) The right breast showing an isoechoic nodule with ill-defined borders with central vascularity. (B) The left breast showing a hypoechoic nodule with irregular margins and no echogenic pseudocapsule. Bilateral MRI (C and D) was performed with bilateral masses showing enhancement and restriction in the diffusion sequence (not shown). A bilateral percutaneous biopsy was performed with typical hyperplasia on the right breast and infiltrating lobular carcinoma on the left breast.

Findings include masses with homogeneous, heterogeneous, or ring enhancement or non-mass lesions with segmental enhancement. A gland in lactation has a rapid physiological enhancement after the administration of contrast medium with an early plateau, associated with the increase in physiological vascularity. These findings should not be confused with malignancy (Figure 3). 

All the figures incorporated in the article have been presented at the European Congress of Radiology 2020 [13].

## Conclusions

PABC should be suspected and studied in patients with a palpable mass during pregnancy, the first year of postpartum or lactation, and not settle for pregnancy changes. Early clinical suspicion and an adequate imaging approach are imperative in order to improve patient outcomes. Prognosis is worse when PABC is associated with lymph node metastasis, larger tumor size, and delayed detection.
